# Substitution of Wheat Flour by Extrudate Orange Peel Dietary Fiber Concentrate in Biscuits: Changes in Mixolab Thermomechanical Behavior, Flour Technofunctionality, and Water Sorption Isotherm Properties

**DOI:** 10.1155/ijfo/1657293

**Published:** 2025-06-19

**Authors:** Luis Eduardo Garcia-Amezquita, Viridiana Tejada-Ortigoza, Mayra Deyanira Ramírez-Aguirre, Alejandra Cantu-Cantu, Esther Pérez-Carrillo, Jorge Welti-Chanes

**Affiliations:** ^1^Escuela de Ingeniería y Ciencias, Tecnologico de Monterrey, Monterrey, Nuevo Leon, Mexico; ^2^Escuela de Ingeniería y Ciencias, Food and Biotech Lab. Tecnologico de Monterrey, Zapopan, Jalisco, Mexico

**Keywords:** adsorption isotherms, dietary fiber concentrate, extrusion, orange peel, technological functionality

## Abstract

Applicability of dietary fiber concentrates (DFCs) from fruit byproducts in food formulations is limited by the high proportion of insoluble dietary fiber (IDF) and its negative effect on sensory and functional quality. Extrusion modifies dietary fiber composition, impacting technological and physiological functionalities. This research evaluated the effect of wheat flour substitution by unprocessed or extruded orange peel DFC (0%–20%) on the properties of biscuits. Composition, Mixolab analysis, dough extensibility, and solvent retention capacity of composite flours were determined. Proximal composition, sensory acceptance, firmness, and water sorption isotherms of biscuits were evaluated, and Brunauer–Emmet–Teller (BET) equation parameters were estimated. Soluble dietary fiber (SDF) and IDF ratio increased with extrusion from 0.15 to 0.98 (w/w). Extruded DFC 5% and 10% substitution had the least detrimental effect on Mixolab profile, extensibility, and solvent retention capacity. The use of unprocessed orange peel decreased technological characteristics. Proximal composition and sensory acceptance had no statistical difference. Extruded DFC increased the high molecular weight SDF content in biscuits. Textural shelf-life of biscuits showed that extruded DFC at 10% substitution generates a biscuit with similar initial hardness with less change during 10 days of storage. Water adsorption isotherms of biscuits with orange peel DFCs showed a direct increase in values according to the substitution in wheat flour. The BET model had a good fitting to describe the adsorption experimental data. The increase in DFC content in the biscuits favored the moisture retention capacity throughout the *aw* range of the isotherms. Extruded orange peel DFC could be used to reduce wheat flour used in biscuit formulations without compromising dough machinability and textural quality, with a positive nutraceutical effect due to the increase in TDF, specifically high-weight soluble dietary fiber.

## 1. Introduction

Nowadays, food options are intended not only to satisfy hunger and to provide the necessary nutrients for humans but also to prevent nutrition-related diseases and improve well-being. To develop these foods, the industry has explored the functional abilities of ingredients such as dietary fiber (DF) [1]. The interest in increasing DF content in commonly consumed foods has risen due to the physiological importance of its consumption. This trend has favored the design of numerous bakery products (bread, cakes, and cookies) with high DF content [2]. DF technological and physiological functionality depends on its soluble and insoluble fractions. For instance, soluble dietary fiber (SDF) is easily solubilized in water and includes pectins, gums, inulin-type fructans, and some hemicelluloses, whereas insoluble dietary fiber (IDF) is not water-soluble and consists of lignin, cellulose, and some hemicellulose [3]. The options for fiber sources in the food industry are extensive. However, in recent days, the interest in obtaining DF raw materials from byproducts has increased due to the change to a circular economy. Orange (*Citrus sinensis)* peel is the main byproduct obtained from the juice processing industry. Due to their chemical composition, orange peels have been used as nonconventional sources of DF. For instance, it has been reported that it has been up to 48.7% (dry basis) of total DF in orange peels [4]. Orange peel DF modifications through processing have also been evaluated. Talens et al. [5] studied the effect of microwave and hot air drying on its physicochemical properties. Tejada-Ortigoza et al. [6] evaluated the effect of high hydrostatic pressure and mild heat treatment on the hygroscopic properties of orange peel DF. García-Amezquita et al. [4] studied the effect of twin extrusion, and no differences were observed between dietary unprocessed fiber (UF) and extruded fiber (EF) concentrates regarding moisture, fat, protein, ash, and total DF content, but with an important increase in terms of SDF. The proximate composition and functional properties dietary UF and EF concentrates from orange peel used were previously analyzed by Garcia-Amezquita et al. [4]. Extrusion generated an increase in SDF (3.7 times), SDFP (high molecular weight soluble) (4.0 times), and technological functionality in terms of solubility (SOL) (15.7%), swelling capacity (SC) (23.6%), and water retention capacity (41.2%) [4]. The effect of extrusion was similar to that reported by Khanpit et al. [7], and the changes may have resulted from the development of porosity, capillary attraction, and break of oligosaccharides structures [3, 8] On the other hand, a decrease could be observed in IDF content of 57.1% and oil retention capacity to 54.4% when compared to the dietary UF concentrate. Changes due to extrusion affect the internal and external structure of fiber and degradation of IDF, such as cellulose and hemicellulose, during the process of sweet potato residues [9].

Bakery products are staple foods that may provide interesting opportunities to deliver health benefits to large populations while also reducing food waste. Biscuits have been consumed as a tasty and convenient delight, but they often lack important nutritional compounds, such as DF or protein [10], making them not suitable for the modern health-conscious population [11]. Biscuits have a pleasant taste and are compact bakery products; they are ideal for facilitating the availability of nutrients. Nowadays, bakery research seeks to make healthier products with enhanced nutritional value, textural quality, and shelf life [12]. Some studies have used orange peel as a DF source in biscuits, evaluating the DF content, quality, and antioxidant properties [13, 14].

Biscuits have been highly appreciated by consumers due to their relatively low cost, convenience, and variety. Another important aspect about biscuits is that they have the longest shelf-life compared with other bakery products, which could be associated with their low water activity. However, one of the most important changes during the storage of biscuits is the loss of texture, which has been associated with water absorption during storage, and this was probably the main limiting factor of shelf-life because [15]. Water plays an important role in the physical stability of food; water sorption isotherms are useful tools that could help to predict changes occurring in the food when exposed to different relative humidity, and parameters obtained from their modeling are useful in the characterization, prediction of the shelf-life, selection of storage conditions, and appropriate packing materials [16]. Furthermore, the moisture sorption isotherms of food provide valuable information for developing new food products and predicting the food shelf-life.

Orange is well known for its antioxidant, anti-inflammatory, and anticarcinogenic properties due to the rich content in Vitamin C, essential oils, phenolics, flavonoids, DFs, etc. Around 70% of the orange is processed in food industries producing juices, jams, marmalades, and essential oils, utilizing only 40%–50% of total mass [17]. Cirrrincione et al. [12] in a review on orange waste revalorization and its application in the bakery industry reported at least six works in the last 5 years about the use of this byproduct in biscuits, and all cases observed an increase in fiber content and other parameters studied were antioxidant effect and minerals. In the case of sensorial characterization, Belose et al. [18], Talents et al. [5], and Teke et al. [19] observed a good acceptance using orange peel fiber concentrate. Interestingly, in the case of the shelf-life of the product, of all works reported, only Belose et al. [18] showed data about sensory, nutritional, and textural changes. However, water sorption isotherm data have not been reported yet, and the effect of DF on water interactions is an important parameter that has not been studied in biscuit products developed using orange peel as a fiber source, as well as textural shelf-life changes associated with this. In addition to this, even though there are some works about the extrusion of orange peel and pulp, the application on biscuits has not been evaluated.

Following the previous work [4], the objective of this study was to compare the difference in wheat flour substitution at different levels by non-EF and EF orange peel in biscuit dough technofunctionality, physicochemical composition, textural shelf-life, and component interactions with water through the study of water absorption isotherms.

## 2. Materials and Methods

### 2.1. Materials

Soft wheat flour (10% protein content, 12% moisture, and 1.5% ash), sweetener (sucralose), baking powder, nonfat dry milk, butter, vanilla extract, and ground cinnamon were purchased from a local market in Monterrey, Nuevo León (Mexico). All chemicals and reagents used were of analytical grade. All reagents for DF quantification were obtained from Sigma-Aldrich (St. Louis, Missouri), and those used for protein and determinations were purchased in DEQ (Monterrey, Mexico). Dietary fiber concentrates (DFCs), UF and EF, were obtained according to Garcia-Amezquita et al. [4]. The EF fiber concentrate used in this work was selected based on the highest SDF content obtained after processing by Garcia-Amezquita et al. [4]. Composite flours were composed of soft wheat flour and the correspondent evaluated substitution of DFC, either UF or EF; the treatments were wheat flour (CC-00) and every composite flour (wheat flour + DFC) with different concentration levels (0%, 5%, 10%, 15%, and 20%) of dietary UF and EF concentrates from orange peel, respectively.

### 2.2. Technological Functionality of DFCs

SOL of the UF and EF concentrates was obtained following the methodology described by Robertson et al. [20] with slight modifications. Samples (200 mg) were suspended in water (30mL) in 50 mL parafilm-covered beakers and stirred for 3 h at 25°C. Suspensions were placed in 50 mL tubes, centrifuged at 3000 × g at 22°C for 20 min. The supernatant was carefully removed, and the pellet was washed with 10 mL of distilled water and filtered using previously weighed filter paper (Whatman No. 41). The pellet was dried at 60°C overnight and weighed (*W*_FP_, g), SOL (%) was calculated following Equation (1), where *W*_*S*_ is the weight of the dry solids of the sample expressed in grams.

(1) $$\begin{array}{c} \text{SOL}=\frac{W_{S}-W_{\text{FP}}}{W_{S}}\times 100. \end{array}$$

SC was obtained by the method reported by Raghavendra et al. [21] with some modifications and is defined as the resulting volume of the sample after hydration with water excess. Briefly, 0.2 g (*W*_*S*_) of each sample was suspended using 10 mL of distilled water in a foil-covered 15-mL graduated test tube and gently stirred with a glass tube. After 24 h at 25°C, the volume of the hydrated precipitate was recorded (*V*_*P*_). SC (mL·g^−1^) was calculated using Equation (2).

(2) $$\begin{array}{c} \text{SC}=\frac{V_{P}}{W_{S}}. \end{array}$$

Water holding capacity (WHC) (mL·g^−1^) is defined as the quantity of water that is retained by the sample after the application of a force, which was attained using the methodology proposed by Chau and Huang [22] with some modifications. Samples (*W*_*S*_ = 1 g) were placed in 50 mL foil-covered beakers with 10 mL of distilled water and continuously stirred for 18 h at 25°C. Suspensions were carefully placed in 15 mL graduated tubes and centrifuged at 4500 × g, 22°C for 30 min. The volume of the supernatant (*V*_*S*_, milliliters) was registered. This property was calculated following (3). Triplicates were performed for each functional property.

(3) $$\begin{array}{c} \text{WHC}=\frac{10-V_{S}}{W_{S}}. \end{array}$$

Oil holding capacity (WHC/OHC, mL·g^−1^), which is defined as the quantity of oil that is retained by the sample after the application of a force, was attained using the methodology proposed by Chau and Huang [22] with some modifications. Samples (*W*_*S*_ = 1 g) were placed in 50 mL foil-covered beakers with 10 mL of soybean oil and continuously stirred for 18 h at 25°C. Suspensions were carefully placed in 15 mL graduated tubes and centrifuged at 4500 × g, 22°C for 30 min. The volume of the supernatant (*V*_*S*_, milliliters) was registered. This property was calculated following (4). Triplicates were performed for each functional property.

(4) $$\begin{array}{c} \text{OHC}=\frac{10-V_{S}}{W_{S}}. \end{array}$$

### 2.3. Biscuit Preparation

Butter (12.4%), nonfat dry milk (9.1%), vanilla extract (8.3%), baking powder (1.7%), sucrose (1.5%), and ground cinnamon (0.8%) were placed in a bowl and mixed (3 min, slow speed) with 49.6% of respective flour for treatment. Then, 16.6% of water (d.b.) was incorporated and mixed at the same speed for 5 min. The cookie dough was sheeted to 5 mm thickness and shaped with a dough cutter (50 mm diameter) and baked at 175°C for 20 min in a toaster oven (GE Model 169104).

### 2.4. Proximate Composition

Moisture, crude protein (*N* × 6.25, for fiber concentrate and 5.7 for biscuits), ash, and fat content were determined for biscuits by employing the standard methods 920.151, 920.152, 940.26, and 960.39, respectively [23]. DF contents were estimated according to Garcia-Amezquita et al. [24] using the AOAC Official Method (2011.25).

### 2.5. Mixolab Analysis, Dough Rheological Properties, and Solvent Retention Capacity (SRC) of the Composite Flours

Mixolab conditions for the study of composite flours were done according to the AACC Method 54-60.01. Mixolab is useful to know how to change process and technological characteristics [25]. Dough extensibility (grams) was evaluated according to the AACC Method 54-21 using the TA.XT2 texture analyzer (TA) equipped with Kieffer rig, at hook speeds 3.3 mm/sec. Dough TPA profile was done at 20 ± 2°C using the same TA. The probe used was a P/75 aluminum, 75 mm diameter, and the test speed was 0.5 mm/s. Three determinations were made. SRC was determined according to AACC Method 65-11. SRC is the weight of solvent held by flour after centrifugation. It is expressed as a percentage of flour weight on a 14% moisture basis. Four solvents are independently used to produce four SRC values: water, aqueous sucrose solution (50%), aqueous sodium carbonate solution (5%), and aqueous lactic acid solution (5%). The combined pattern of the four SRC values establishes a practical flour quality/functionality profile useful for predicting baking performance and specification conformance. Generally, lactic acid SRC is associated with glutenin characteristics, sodium carbonate SRC with levels of damaged starch, and sucrose SRC with pentosan characteristics.

### 2.6. Sensory Analysis

A hedonic test was performed to determine the degree of sensory acceptance of the biscuits. A 1–5 scale was used, where 1 and 5 were the least and most accepted values, respectively. Fifty untrained panelists between 19 and 38 years old evaluated texture, flavor, and overall acceptance of each sample.

### 2.7. Moisture Sorption Isotherms

Biscuit samples were milled using a mortar and pestle and sieved through a mesh number 40 (425 *μ*m). The samples were stored in desiccators containing P_2_O_5_ (25°C) for at least 5 days before analysis. Initial moisture content was determined as specified by AOAC Official Method (920.151). Dynamic dewpoint isotherms (DDIs) were obtained for each treatment using the Aquasorp Isotherm Generator manufacturer's instructions (Decagon Devices Inc., Pullman, Washington). Duplicate adsorption moisture isotherms in the ~0.10–0.93 *a*_*w*_ range were determined at 30°C with ± 0.0001 *a*_*w*_ resolution. The BET isotherm model was applied to fit the experimental data in the interval of 0.1–0.5 *a*_*w*_, and the model parameters (monolayer moisture and constant *C*) were determined [26].

### 2.8. Texture

The firmness of the biscuits during storage (10 days a room temperature) was measured using a TA (TA-XT2 Stable Micro Systems Ltd.). Firmness (grams) is defined as the maximum force on a product that displays substantial resistance to deformation and was evaluated using a cylinder probe (diameter 3 mm) in triplicates.

### 2.9. Statistical Analysis

Minitab Statistical Software v.18 (Minitab Inc., State College, Pennsylvania, United States) was used to analyze the data with ANOVA to determine whether there were statistically significant differences among three replicate means.

## 3. Results and Discussion

### 3.1. UF and EF Concentrate Characterizations

Table 1 shows the proximate composition and functional properties of UF and EF concentrates from orange peel. No differences were observed between UF and EF in terms of moisture, crude fat, crude protein, ash, and total DF content. Extrusion processes provoked an increase in the content of SDF (3.7 times) and SDFP (4.0 times), phenomena that could be associated with the breakdown of glycosidic bonds of IDF into smaller soluble fractions [3]. Extrusion generated a change in the technological functionality of DFCs, increasing SOL (15.7%), SC (23.6%), and water retention capacity (41.2%). Similar effects of extrusion were reported by Khanpit et al. [7]. These changes may have resulted from the development of porosity, capillary attraction, and hydrophilia [8] and the break of oligosaccharides [3, 8] On the other hand, a decrease was observed in IDF content of 57.1% and oil retention capacity to 54.4% when compared to the dietary UF concentrate.

### 3.2. Mixolab Analysis and Dough Texture Properties

Table 2 shows the results of dough rheology and texture analysis. Substitution of wheat flour by different UF and EF generated differences in the viscoelastic properties of dough (Table 2). In the first stage of the Mixolab cycle, viscoelastic properties, including hydration and mixing tolerance index, were evaluated. Maximum dough consistency at initial mixing (C1) values were targeted to 1.1 N·m according to AACC Method 54-60.01. Dough stability decreased with a higher substitution of EF (higher DF content); dough formulated with UF showed a clear decrease concerning the control, 4.81 and 9.20 min, respectively. However, there was no relation between DF content and stability values. Schmiele et al. [27] did not observe the difference caused by the addition of inulin in wheat flour for bread production in the first stage curve. The differences observed in dough stability when comparing control with the flours containing UF and EF could be associated with the technological functionality properties (SOL, SC, and water retention capacity) of the concentrates [4]. According to Li et al. [28], the effect of hydrocolloids in dough would be attributed partially to the interactions between gluten protein and nonionic hydrocolloids through hydrogen bonding and electrostatic interactions. In the case of pectin, in addition to a dilutive effect on gluten, it could establish electrostatic interactions with gluten proteins, whereas a repulsive effect might also exist between contiguous chains showing a more filamentous structure and causing disaggregation of the gluten network. Results showed that control is an intermediate flour, taking the reference values reported by Dubat et al. [29]. This type of wheat flour is adequate to produce this type of biscuit. The parameter C2 is related to protein weakening based on mechanical work and temperature increase, and no significant difference was observed between the control and dough with EF; the highest difference was obtained between the control (0.44 Nm) and UF-15 composite flour (0.29 Nm) (Table 2). Similar results were observed in flours with DF at different concentration levels, where the C2 reduction could be attributed to the interaction between flours with a major content of IDF with the protein unfolding [30]. In the case of parameter C3, related to starch gelatinization, the use of UF or EF at levels of 5% and 10% does not affect this parameter. However, an important C3 value reduction (0.22 Nm) was observed on UF 15, 20, EF 15, and 20 at 15% substitution. A synergistic thickening effect was seen when starch granules were further in the continuous phase containing hydrocolloids [28]. The stability of the hot-formed gel (C4) and starch retrogradation during the cooling period (C5) were affected in all the studied samples. Treatment EF-5 (*C*4 = 1.69 Nm; *C*5 = 2.69 Nm) had the lowest effect, and the treatments with the highest effect, in both parameters, were UF-20 (*C*4 = 1.24 Nm; *C*5 = 1.87 Nm) and EF-15 (*C*4 = 1.30 Nm; *C*5 = 1.88 Nm). The effect in C4 could be due to the dilution of wheat flour starch and the complex formed by starch and fiber through hydrogen bonds and van der Waals forces, which changed the viscosity and expansion of starch [11]. Changes in C5 are related to the interaction with solubilized amylose and its recrystallization, meaning that the addition of DF to the samples could inhibit the reassociation of amylose chains due to this interaction. This antiretrogradation effect on starch may be important to prevent stalling in bakery products. During heating and cooling stages, the control flour showed a high viscosity compared with wheat flour control (CC) due to starch gelatinization and retrogradation, as expected according to Schmiele et al. [27].

Dough extensibility showed an interesting behavior with the highest increase (28.82 mm) at 10% EF substitution and showed a relation with C2 values reported for different flours. In the case of dough with UF, the effect on extensibility was observed at substitution levels of 15% and 20%. However, in the case of dough with EF at all substitution levels, an increase in C2 values was observed. Šoronja-Simović et al. [31] observed a similar effect on extensibility using carob pod flour and sugar beet fiber. The extensibility of wheat flour dough is related to gluten development, and nonstarch polysaccharides play important roles in the functionality of the same. SDF interacts with gluten noncovalently via hydrogen bonding and hydrophobic interactions [32]. Molecules of SDF limit the water availability in dough for the other biopolymers as reflected in more *β*-sheet structures, reduced water mobility, and lower FW content. The reduction in water availability results in impaired development of an elastic gluten network and an increased resistance to deformation [33]. In the case of TPA, dough profile hardness and chewiness increase with the level of substitution, with the highest values of 537.6 g of hardness and 59.07 g for adhesiveness, for samples EF-20. Interestingly, in the case of hardness, UF substitution also affects the dough hardness but with less intensity than in EF substitution. Wang et al. [34, 35] observed that IDF added to wheat flour increases the mechanical resistance of the dough and associated this effect with the interaction between IDF and gluten that reduces the plasticising effect of water due to the strong water absorption and filling effect. DF makes part of water unavailable for the dough creation; in the case of insoluble dietary, a loss of dough characteristics was expected, but we can observe that an increase in SDF gives support for dough, which could even be harder.

No differences were found among cohesiveness with EF substitution; however, with UF, a decrease of 0.15 g was observed. Springiness was reduced by 0.16% with the increase of DF content compared to the control (Table 2). Peressini and Sensidoni [36] studied the effect of using inulin with different degrees of polymerization on wheat flour dough, and they reported that the addition of DF might have altered the ionic and hydrophobic interactions and covalent and hydrogen bonds, avoiding full hydration of the gluten protein and dilute protein content, additionally forming a matrix to hold the starch. A similar effect was observed in the present work, considering DF composition and its SOL. It is well known that the rheological characteristics of dough are critical and that they affect handling and processing. In industrial and commercial processing, including fiber and other nutraceuticals, it is a challenge, considering that the inclusion affects dough rheology, which will not result in a satisfactory product. The EF that was evaluated in this work proved to be a suitable alternative, considering that it has a minimum negative effect on the biscuit dough used in its model. Experimental composite flour EF-10 is a promising alternative considering this aspect and a possible reduction of retrogradation. Mixolab and TPA analysis could give important information to estimate the performance of new formulations and considerations to adjust water addition and time of dough processing.

### 3.3. SRC

SRC was affected by the DF substitution (Table 2). In the case of water and sucrose solutions, significant differences were found with substitution levels of 15% and 20%. Water and sucrose solution retention capacity increased from 60.7% in the control to 72.8% and from 96.6% to 101.7%, respectively, with a UF substitution of 5%. Valerga et al. [37] observed a similar effect on wheat flour SRC when using artichoke, eggplant, and tomato flour to substitute wheat flour. For lactic acid retention capacity, significant differences were found in EF-05 and EF-20 compared to CC-00. In the case of EF-05, there was a reduction of 32.29%; in contrast, an increment of 30.3% was found with EF-20. Ahmad et al. [38] observed a similar effect for carrot pomace powder at 15% and 20% substitution, attributing the result to the dilutive effect. In general, the retention capacity of lactic acid is associated with the formation of glutenin network and the strength of gluten in flour. The stabilization of this protein network is mediated by the presence of soluble polymeric carbohydrates, such as pentosans, favoring the formation of macro peptides. This result suggests that there is an interaction between SDF of EF, specifically due to the increase on SDFP fraction (Table 1), considering that this fraction is composed of arabinoxylan (pentosan present wheat flour), fructan, and glucans that could increase the strength of the gluten noncovalently via hydrogen bonding and hydrophobic interactions altering secondary and tertiary structures, disulfide bridges, and hydrogen bonding patterns [32]. This study on SRC showed how using this new raw material could change techno-functionality. The use of 10% of EF did not affect SRC capacity, and formulation has not been changed to preserve biscuit characteristics.

### 3.4. Biscuit's Composition and Sensory Acceptance

During dough preparation, the water content was the same for all samples, which is reflected in the final product moisture, where no significant differences could be observed (Table 3). Statistical differences were not observed in terms of protein and fat content. Similar results were observed by Ramashia et al. [39], Elgindy [40], and Magda et al. [41] in the use of orange peel flours on different biscuit formulations. As expected, a clear relation between the increase in UF and EF levels and TDF increase content could be observed in Table 3. However, a null effect on TDF concentration in the ending product was observed in 5% substitution, which could be explained due to the slight difference in EF and UF TDF content (48% and 49%, respectively, Table 1). UF-20 and EF-20 had 2 and 1.8 times more TDF content than the control biscuit, respectively. IDF content increased 4.6 times in UF-20 and 2.6 times in EF-20 compared with the control, meaning an increase of 1.8 times between EF-20 and UF-20, which is attributed to the IDF composition of dietary UF and EF concentrates. Rani et al. [42] observed a similar effect on TDF content due to the addition of orange peel powder. Sensory analysis showed that consumers did not have a preference in terms of biscuit texture due to the different treatments (Table 3). However, in terms of flavor, UF-20 had less acceptability than EF-5, EF-10, and CC-00; this difference could be associated with the effect of extrusion on the fiber structure. In research by Marzec et al. [43] with wheat and oat fiber use in shortbread biscuits, it was observed that the biscuit with oat fiber sensory acceptance sweet flavor was highest, and this could be associated with SDF content. For overall acceptance, CC-00, UF-05, and EF-10 had no significant differences, while UF-10 was the least accepted.

### 3.5. Biscuit's Instrumental Texture

Biscuits textural parameters are important to consumers. Parameters obtained in this work are in the values reported for this kind of product [44, 45]. One of the most important quality deterioration phenomena in biscuits is the loss of crispness due to moisture uptake. Hardness in biscuits formulated with UF and EF decreased with the substitution of DF, except in UF-10 (Table 4). The highest difference was found between the control and UF-20. The decrease in hardness could be due to the competition of EF and UF and flour proteins for water, which results in a lack of gluten development. Mixolab parameter C2 confirmed this possible effect (Table 2). On the other hand, initial *a*_*w*_ values (Table 4) are different between UF treatments and all others, and this could be due to the plasticization of the material by water [46]. Similar results in terms of biscuit texture were reported by Ahmad et al. [38] using carrot pomace powder when using UF. Lee and Inglett [47] observed a similar effect on cookies due to the partial substitution of fat by jet cooker treated oat bran. During the storage of UF biscuits, the hardness remained and, in some cases, increased to the value at day 0 (Table 5), and the control hardness was reduced to half. These differences were attributed to the starch–fiber interaction with wheat. Biscuits produced with EF substitutions had fewer changes in terms of hardness. In Table 3, we could observe that EF substitution at the 20% level duplicates the level of SDF and could partially explain textural shelf-life stability, considering that, according to Huang et al. [48], some of these components can form gels with water.

### 3.6. Moisture Sorption Isotherms

**Figure 1:**
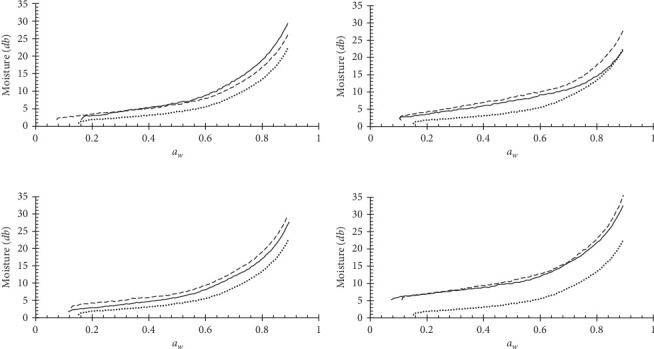
Adsorption isotherms at 30°C of low-caloric biscuits formulated with different wheat flour substitution levels ((a) 5%; (b) 10%; (c) 15%; (d) 20%) using raw- (----) and extruded-dietary fiber concentrate (—) from orange peel compared with the control (·····).

The experimental sorption isotherms data obtained with DDI method at 30°C for biscuits produced from all treatments (control, with UF and EF at 5%, 10%, 15%, and 20% substitution in wheat flour) are shown in Figure 1. In all cases, it can be noted that all samples had the form of Type III isotherm according to the BET classification [49, 50]. Foods with high content of soluble components commonly show this form [51]. Above 0.75 *a*_*w*_, the curves showed a steep rise attributable to the dissolution of sugars in the product aqueous phase [52, 53]. The isotherm obtained in this study without the incorporation of the fiber concentrate is very similar to those obtained by Wang et al. [34, 35] for biscuits made with whole-grain wheat at different initial temperatures and moisture. As can be seen, no important differences are detected between the isotherms of the samples with the EF or non-EF material. However, the isotherms of the samples to which the orange peel was not added are located below the isotherms of the products that have any UF or EF. This could indicate that this product has a lower moisture retention capacity and, therefore, could be more unstable during storage. With the maximum substitution level (20%), it can be observed a clear difference when compared to the control sample, increasing the moisture sorption content ≈2 times its original value through all the evaluated *a*_*w*_ range. The effect of the increase in equilibrium moisture content for the samples with UF and EF at the given *a*_*w*_ generates products with a potential longer shelf-life. This change due to composition could be explained by fiber interaction with water, generating a reduction in molecular motion, and as a result, the products could be more stable. Similar results were also reported for cookies by McMinn et al. [54] and Sampaio et al. [55]. It was expected, considering that the major component of the biscuit's formulation is soft wheat flour and the adsorption properties of this material depend on its interactions with the water molecules, being starch the main component involved in this process [56]. However, the impact of fiber concentrates in the adsorption process, even at low levels of substitution, is evident. At all substitution levels, both biscuits formulated with raw and EF DF concentrates increased their sorption capacities when compared to the control sample. Panjagari et al. [57] observed a similar effect on biscuits due to the addition of beta-glucans, and this property was attributed to fiber.  Table 4 shows that the BET model adequately describes the moisture sorption isotherms of all the products under study by obtaining correlation coefficients greater than 0.979. The monolayer moisture or maximum stability moisture at 30°C for the control biscuits was 2.36% (db), while for products with 20% UF and EF concentrates, the values were 5.47% and 5%, respectively. The values obtained here are similar to those also reported by Sampaio et al. [55] for biscuits formulated with wheat, oatmeal, and passion fruit, with monolayer moisture in the order of 6.08% (db) at 25°C. On the other hand, in a study conducted by Romani et al. [50] in which they evaluated the effect of biscuit storage time on moisture sorption isotherms at 25°C, BET monolayer moisture values between 1.473% and 2.08% (db) are reported, which are very similar to the monolayer value of biscuits without the addition of fiber concentrate. As can be seen in Table 4, regarding the constant *C* of the BET model, which is related to the binding energy of water to the dry matter of the food, or net heat of sorption, it generally increases with increasing fiber content in biscuits. The lowest value of *C* is 6.12 and occurs in the sample without incorporated fiber, while the highest values are 182.8 and 62.56 for the products with the highest levels of fiber (20%), non-EF and EF, respectively. In the study conducted by Panjagari et al. [57] to evaluate the sorption isotherms of beta-glucan rich composite flour biscuits at 28°C, 37°C, and 45°C, the *C* values obtained by BET model were between 2.75 and 6.24 and 14.016 to 38.287 with the GAB model, while in the study by Sampaio et al. [55], the *C* values were between 8.99 and 40.503. The increase in *C* values reflects a better capacity to retain water and is appropriately related to the monolayer moisture values obtained. To the extent that the monolayer moisture values are lower, it can be inferred that it is necessary to reduce the final moisture of the product to lower levels so that it is stable during storage at the temperature at which the isotherm was obtained [49]. These results indicated that products formulated with fiber have a higher capacity to retain water and make it less available for deterioration during storage. It is important to note that, in general, monolayer moisture values are higher for the product formulated with non-EF fiber than those with EF fiber, which is related to the higher content of low molecular weight carbohydrates (soluble fiber) in the latter products. The *a*_*w*0_ values calculated with the BET model using the monolayer moisture value were lower in the products with added fiber in all samples, with 0.11 and 0.06 being the lowest values obtained with the products formulated with the incorporation of non-EF and EF fiber. Lower *a*_*w*_ values reflect the great water retention capacity of incorporating the highest levels of fiber, even though the moisture levels are the highest.

## 4. Conclusion

No differences (*p* < 0.05) were observed in moisture, protein, fat, ash, TDF, and SDFS content between the UF and EF orange peel DFC. The SDF content was directly affected by the SDFP fraction, with an increase of 3.7 times in dietary EF concentrate compared with dietary non-EF concentrate. Concerning dough elaborated with UF and EF concentrates, the Mixolab profile reflected that substitution with EF DF did not affect protein interactions, while the stability of gel and starch retrogradation was negatively affected. Moisture, protein, and ash composition of biscuits with and without DF did not present significant differences; nevertheless, TDF content increased. Biscuits with EF substitution reflected a clear increment in SDF content, where the highest increment observed was 47% between EF-20 and the control. The extrusion had a positive effect on biscuit characteristics, considering DF increase, improving water retention capacity. EF orange peel DFC may be used to substitute 10% of the wheat flour used in biscuit formulations without compromising the product quality in terms of dough machinability and *a*_*w*_, while improving water retention capacity and the increase of SDF content. The BET model was found suitable to describe the sorption isotherms of the formulated products, and the values obtained from the monolayer moisture and the constant *C* of the model indicate the favorable effect of the incorporation of the fiber present in the orange peel. DF components present in the orange peel concentrate increased monolayer moisture content, which represents a biscuit rich in DF that could be a good alternative to extended shelf-life products like biscuits. The high content of high-weight SDF fraction represents a possible beneficial effect on gut microbiota and, therefore, on health.

## Figures and Tables

**Table 1:** Proximate composition and functional properties of dietary unprocessed fiber (UF) and extruded fiber (EF) concentrates from orange peel. Composition values are expressed in g∙100 g^−1^ db.

	**UF**	**EF**
Moisture	2.2 ± 0.1^a^	2.1 ± 0.1^a^
Crude fat	1.5 ± 0.1^a^⁣^∗^	1.4 ± 0.1^a^
Crude protein	4.9 ± 0.1^a^⁣^∗^	4.8 ± 0.0^a^
Ash	4.2 ± 0.1^a^⁣^∗^	4.1 ± 0.1^a^
Total dietary fiber (TDF)⁣^∗∗^	49.2 ± 0.0^a^⁣^∗^	48.2 ± 0.5^a^
Insoluble dietary fiber (IDF)	42.7 ± 0.5^a^⁣^∗^	24.4 ± 0.2^b^
Soluble dietary fiber (SDF)⁣^∗∗∗^	6.4 ± 0.3^b^⁣^∗^	23.8 ± 0.1^a^
SDFP	5.6 ± 0.2^b^⁣^∗^	22.4 ± 0.0^a^
SDFS	0.9 ± 0.0^a^⁣^∗^	1.3 ± 0.3^a^
SDF/IDF	0.15 ± 0.01^b^	0.98 ± 0.01^a^
SDF/TDF	0.13 ± 0.01^b^	0.49 ± 0.00^a^
Solubility (%)	44.7 ± 1.0^b^	51.7 ± 0.8^a^
Swelling capacity (mL·g^−1^)	10.6 ± 0.3^b^	13.1 ± 0.5^a^
Water retention capacity (mL·g^−1^)	3.4 ± 0.1^b^	4.8 ± 0.1^a^
Oil retention capacity (mL·g^−1^)	2.5 ± 0.1^a^	1.36 ± 0.0^b^
pH	5.29 ± 0.01^b^	5.32 ± 0.02^a^
Tapping density (g·mL^−1^)	0.45 ± 0.01^b^	0.82 ± 0.00^a^

*Note:* Mean value of three determinations ± SD. Different letters in the same row are significantly different (*p* < 0.05). SDFP: high molecular weight soluble dietary fiber; SDFS: low molecular weight soluble dietary fiber.

⁣^∗^Data obtain from Garcia-Amezquita et al. [4].

⁣^∗∗^TDF was obtained as the sum of IDF and SDF.

⁣^∗∗∗^SDF as the sum of SDFP and SDFS.

**Table 2:** Mixolab analysis, dough texture properties and solvent retention capacity of composite flours without (CC-00) and with different concentration levels (5%, 10%, 15%, and 20%) of dietary unprocessed fiber (UF) and extruded fiber (EF) concentrates from orange peel.

	**CC-00**	**UF-05**	**UF-10**	**UF-15**	**UF-20**	**EF-05**	**EF-10**	**EF-15**	**EF-20**
*Mixolab profile*									
Stability (min)	9.20 ± 0.14^a^	5.60 ± 0.11^c,d^	4.81 ± 0.44^d^	5.92 ± 0.78^c,d^	5.34 ± 0.12^d^	7.64 ± 0.13^b^	7.00 ± 0.89^b,c^	5.07 ± 0.42^d^	5.30 ± 0.07^d^
C1 (N·m)	1.12 ± 0.01^a^	1.11 ± 0.04^a^	1.13 ± 0.04^a^	1.02 ± 0.00^a^	1.05 ± 0.14^a^	1.15 ± 0.01^a^	1.12 ± 0.06^a^	1.03 ± 0.05^a^	1.12 ± 0.03^a^
C2	0.44 ± 0.00^a^	0.37 ± 0.01^b,c,d^	0.32 ± 0.01^c,d,e^	0.29 ± 0.00^e^	0.30 ± 0.05^d,e^	0.42 ± 0.00^a,b^	0.41 ± 0.02^a,b^	0.38 ± 0.02^a,b,c^	0.44 ± 0.01^a,b^
C3	1.77 ± 0.00^a,b^	1.72 ± 0.04^a,b,c^	1.68 ± 0.00^b,c,d^	1.61 ± 0.00^d,e^	1.58 ± 0.05^d,e^	1.78 ± 0.01^a^	1.73 ± 0.03^a,b^	1.55 ± 0.01^e^	1.63 ± 0.02^c,d,e^
C4	1.89 ± 0.03^a^	1.59 ± 0.00^c^	1.46 ± 0.01^d^	1.28 ± 0.00^e^	1.24 ± 0.03^e^	1.69 ± 0.01^b^	1.58 ± 0.03^c^	1.30 ± 0.00^e^	1.42 ± 0.01^d^
C5	3.45 ± 0.02^a^	2.52 ± 0.04^c^	2.20 ± 0.01^e^	2.01 ± 0.00^f^	1.87 ± 0.04^g^	2.67 ± 0.00^b^	2.39 ± 0.03^d^	1.88 ± 0.00^g^	2.05 ± 0.03^f^

*TPA dough profile*									
Hardness (g)	131.6 ± 8.4^f^	250.0 ± 15.0^e^	346.8 ± 28.9^c,d^	367.4 ± 35.1^b,c^	253.4 ± 18.1^e^	309.0 ± 16.2^d^	319.4 ± 13.4^d^	404.8 ± 11.7^b^	537.6 ± 15.5^a^
Springiness (%)	0.41 ± 0.01^a^	0.34 ± 0.03^b^	0.32 ± 0.02^b,c,d^	0.28 ± 0.03^c,d,e^	0.24 ± 0.00^e^	0.33 ± 0.07^b,c^	0.32 ± 0.05^b,c,d^	0.27 ± 0.04^d,e^	0.25 ± 0.06^e^
Cohesiveness	0.40 ± 0.01^a^	0.37 ± 0.02^a^	0.30 ± 0.03^b^	0.29 ± 0.05^b,c^	0.25 ± 0.02^c^	0.33 ± 0.07^a,b^	0.28 ± 0.05^b,c^	0.28 ± 0.04^b,c^	0.28 ± 0.07^b,c^
Adhesiveness (g)	22.62 ± 1.57^d^	18.00 ± 1.00^d.e,f^	27.51 ± 1.35^c^	34.05 ± 2.57^b^	14.51 ± 1.19^f^	17.38 ± 1.60^e,f^	19.91 ± 2.38^d,e^	22.51 ± 1.76^d^	59.07 ± 7.67^a^

*Dough extensibility*									
Distance (mm)	23.04 ± 0.93^e^	22.04 ± 1.40^e^	20.70 ± 0.76^e^	37.25 ± 0.92^c^	30.35 ± 21.05^d^	43.18 ± 2.90^b^	51.85 ± 2.55^a^	41.08 ± 2.64^b^	32.55 ± 2.82^d^

*Solvent retention capacity*									
Water	60.7 ± 0.0^e^	72.8 ± 0.4^c,d,e^	65.1 ± 0.1^d,e^	81.2 ± 2.4^b,c,d^	91.8 ± 1.6^a,b^	73.8 ± 0.7^b,c,d,e^	69.6 ± 1.4^d,e^	88.9 ± 7.7^a,b,c^	105.9 ± 10.5^a^
Lactic acid	92.9 ± 0.3^b,c^	84.1 ± 5.3^b,c,d^	78.6 ± 0.5^c,d^	93.8 ± 4.0^b,c^	106.3 ± 2.4^a,b^	62.9 ± 2.8^d^	87.3 ± 4.6^c,d^	105.2 ± 4.0^a,b^	121.1 ± 13.6^a^
Sucrose	96.9 ± 2.9^e^	118.1 ± 2.9^b,c,d^	102.9 ± 0.2^d,e^	123.0 ± 3.0^a,b,c^	139.2 ± 4.5^a^	101.7 ± 2.0^d,e^	114.7 ± 1.0^c,d,e^	129.7 ± 3.2^a,b^	133.6 ± 12.8^a^
Carbonate	72.3 ± 1.5^c^	122.5 ± 0.5^b^	127.7 ± 5.8^b^	151.9 ± 1.4^a^	167.0 ± 14.3^a^	104.7 ± 1.4^b^	120.4 ± 2.5^b^	152.3 ± 0.4^a^	159.5 ± 6.2^a^

*Note:* CC-00: control flour (soft wheat flour). Values are expressed as percentage in wb. Values are an average of at least four determinations ± SD. Means with different letters in each row are statistically different (*p* < 0.05). (C1) Water absorption, (C2) protein weakening as a function of mechanical work and temperature, (C3) starch gelatinization, (C4) stability of the hot-formed gel, and (C5) starch retrogradation during the cooling period.

**Table 3:** Proximate composition and sensorial acceptance of biscuits formulated with different wheat flour substitution levels (5%, 10%, 15%, and 20%) using dietary unprocessed fiber (UF) and extruded fiber (EF) concentrates from orange peel. Composition values are expressed in g∙100 g^−1^ db, except for moisture (wb).

	**CC-00**	**UF-05**	**UF-10**	**UF-15**	**UF-20**	**EF-05**	**EF-10**	**EF-15**	**EF-20**
*Proximate composition*									
Moisture	5.8 ± 1.7^a^	3.3 ± 1.2^a^	3.7 ± 4.0^a^	2.7 ± 1.2^a^	6.9 ± 4.5^a^	2.1 ± 2.0^a^	3.2 ± 0.1^a^	3.5 ± 2.5^a^	3.6 ± 1.4^a^
Crude fat	16.2 ± 0.5^b^	16.3 ± 1.4^a,b^	17.4 ± 0.8^a,b^	15.9 ± 1.3^b^	18.6 ± 1.4^a^	17.0 ± 0.1^a,b^	17.6 ± 1.0^a,b^	15.9 ± 1.7^b^	17.8 ± 0.5^a,b^
Crude protein	13.9 ± 0.8^a,b^	14.1 ± 0.1^a^	11.2 ± 0.2^a,b^	11.3 ± 0.7^a,b^	11.6 ± 0.6^a,b^	12.9 ± 1.7^a,b^	10.5 ± 0.5^b^	11.7 ± 1.4^a,b^	11.5 ± 1.9^a,b^
Ash	2.8 ± 0.3^a^	3.3 ± 0.1^a^	3.0 ± 0.7^a^	3.0 ± 0.6^a^	3.3 ± 0.3^a^	3.2 ± 0.0^a^	3.5 ± 0.1^a^	3.5 ± 0.0^a^	3.5 ± 0.3^a^
*Dietary fiber*									
TDF	8.8 ± 0.4^d^	9.5 ± 0.9^d^	12.9 ± 0.5^c^	14.8 ± 0.0^b,c^	19.2 ± 0.2^a^	9.7 ± 0.8^d^	14.1 ± 0.4^b,c^	14.1 ± 1.0^b,c^	15.8 ± 0.4^b^
IDF	2.4 ± 0.3^e^	3.9 ± 0.4^d^	6.5 ± 0.2^b,c^	7.2 ± 0.9^b^	11.2 ± 0.4^a^	4.0 ± 0.8^d^	5.7 ± 0.1^c^	6.2 ± 0.2^b,c^	6.4 ± 0.2^b,c^
SDF	6.3 ± 0.7^b,c,d^	6.1 ± 0.9^c,d^	6.3 ± 0.5^c,d^	6.7 ± 0.5^b,c.d^	7.6 ± 0.2^a,b,c^	5.8 ± 0.2^d^	8.5 ± 0.4^a^	8.0 ± 0.7^a,b^	9.3 ± 0.3^a^
SDFP	2.5 ± 0.4^d^	3.1 ± 0.7^c,d^	3.3 ± 0.4^c,d^	3.5 ± 0.7^b,c.d^	4.6 ± 0.2*a*^,b^	2.7 ± 0.3^c,d^	4.2 ± 0.1^a,b,c^	4.8 ± 0.3^a,b^	5.6 ± 0.7^a^
SDFS	3.5 ± 0.3^a^	2.5 ± 0.4^a^	2.7 ± 0.7^a^	3.0 ± 0.6^a^	3.1 ± 0.0^a^	2.7 ± 0.3^a^	2.8 ± 0.3^a^	3.1 ± 0.6^a^	3.5 ± 0.3^a^
*Sensory acceptance*									
Texture	3.3 ± 1.1^a,b^	3.4 ± 1.1^a^	2.6 ± 1.2^b^	3.2 ± 1.2^a,b^	3.3 ± 1.0^a,b^	3.3 ± 1.1^a,b^	3.5 ± 1.2^a^	3.3 ± 1.0^a^	3.0 ± 1.0^a,b^
Flavor	3.4 ± 1.3^a^	3.3 ± 1.1^a,b,c^	2.6 ± 1.0^b,c^	2.9 ± 1.2^a,b,c^	2.6 ± 1.2^c^	3.0 ± 1.1^a,b,c^	3.4 ± 1.1^a^	3.1 ± 1.1^a,b,c^	2.7 ± 1.1^a,b,c^
Overall accepting	3.3 ± 1.0^a^	3.3 ± 1.0^a^	2.6 ± 1.1^b^	2.9 ± 1.1^a,b^	2.7 ± 1.0^a,b^	3.1 ± 1.0^a,b^	3.2 ± 1.1^a^	3.1 ± 1.0^a,b^	2.8 ± 1.0^a,b^

*Note:* In case of proximate composition and dietary fiber data, values are the mean of triplicates ± SD. Means with different letters in each row are statistically different (*p* < 0.05). SDFP: high molecular weight soluble dietary fiber, SDFS: low molecular weight soluble dietary fiber. For sensory acceptance, values are the mean of 50 observations ± SD. Means with different letters in each column are statistically different (*p* < 0.05).

Abbreviations: IDF: insoluble dietary fiber, SDF: soluble dietary fiber, TDF: total dietary fiber.

**Table 4:** BET estimated parameters for adsorption isotherms (*a*_*w*_ = 0.05–0.5) of biscuits formulated with different wheat flour substitution levels (5%, 10%, 15%, and 20%) using dietary unprocessed fiber (UF) and extruded fiber (EF) concentrates from orange peel.

	**CC-00**	**UF-05**	**UF-10**	**UF-15**	**UF-20**	**EF-05**	**EF-10**	**EF-15**	**EF-20**
*BET parameters*									
*M*_0_	2.36	3.39	4.84	3.63	5.47	3.87	4.20	2.93	5.00
*C*	6.12	18.09	9.69	49.25	182.80	6.88	11.66	12.88	62.56
*a*_*w*0_	0.28	0.19	0.23	0.15	0.11	0.27	0.23	0.25	0.06
*R* ^2^	0.979	0.997	0.992	0.994	0.996	0.979	0.995	0.971	0.995

*Note: M*_0_ monolayer moisture units are %moisture on a dry basis: *a*_*w*0_ is the water activity at the monolayer moisture value.

**Table 5:** Textural changes through storage (0–10 days) of biscuits formulated with different wheat flour substitution levels (5%, 10%, 15%, and 20%) using dietary unprocessed fiber (UF) and extruded fiber (EF) concentrates from orange peel.

**Treatment**	**Time (days)**				
	**0**	**1**	**4**	**7**	**10**
	**Hardness (g)**				
CC-00	55.3 ± 5.9^a,A^	37.2 ± 0.7^b,c,B^	35.7 ± 1.6^a,b,B^	20.1 ± 0.7^e,f,C^	29.6 ± 1.8^c,d,B^
UF-05	35.4 ± 0.1^c,d,A,B^	40.4 ± 7.8^a,b,A^	23.5 ± 0.9^d,e,B,C^	24.2 ± 2.5^c,d,e,B,C^	15.1 ± 2.9^d,C^
UF-10	46.0 ± 1.4^a,b,A^	46.1 ± 1.3^a,b,A^	29.6 ± 0.7^b,c,d,B^	47.7 ± 2.3^a,A^	34.1 ± 8.0^b,c,d,A,B^
UF-15	31.8 ± 3.6^c,d,B^	35.1 ± 0.9^b,c,B^	41.5 ± 1.0^a,B^	37.4 ± 1.5^b,B^	70.3 ± 6.7^a,A^
UF-20	13.8 ± 0.2^e,C^	9.9 ± 0.6^d,C^	24.5 ± 0.6^d,e,B,C^	28.0 ± 0.8^c,d,B,C^	52.4 ± 10.2^a,b,A^
EF-05	25.2 ± 2.2^d,A^	25.1 ± 4.5^c,d,A^	25.5 ± 0.1^c,d,e,A^	22.4 ± 1.4^d,e,f,A^	26.1 ± 2.2^d,A^
EF-10	41.5 ± 2.0^b,c,B^	53.1 ± 1.2^a,A^	31.4 ± 4.2^b,c,C^	29.4 ± 1.8^c,C^	49.7 ± 1.0^a,b,c,A,B^
EF-15	35.3 ± 1.2^c,d,A^	23.4 ± 6.9^c,d,A,B^	22.7 ± 0.4^e,A,B^	17.1 ± 1.8^f,B^	21.5 ± 3.9^d,A,B^
EF-20	26.8 ± 1.6^d,B^	35.8 ± 0.6^b,c,A^	26.5 ± 1.0^c,d,e,B^	26.1 ± 0.1^c,d,B^	26.9 ± 0.4^d,B^

*Note:* Values are the mean of at least four observations ± SD. Means with different capital letters in each row are statistically different (*p* < 0.05). Means with different lowercase letters in each column are statistically different (*p* < 0.05).

## Data Availability

Data is available on request from the authors.

## General Statement

*Practical Applications*. Wheat flour could be substituted at 10% by extruded orange peel DFC without change in process and improving product stability and soluble dietary content.
